# Extensive Intracranial Meningioma With Dehiscences: A Case Report

**DOI:** 10.7759/cureus.51596

**Published:** 2024-01-03

**Authors:** Corneliu Toader, Luca-Andrei Glavan, Razvan-Adrian Covache-Busuioc, Bogdan-Gabriel Bratu, Horia-Petre Costin, Antonio-Daniel Corlatescu, Alexandru Vladimir Ciurea

**Affiliations:** 1 Department of Neurosurgery, “Carol Davila” University of Medicine and Pharmacy, Bucharest, ROU; 2 Department of Neurosurgery, National Institute of Neurology and Neurovascular Diseases, Bucharest, ROU; 3 Neurosurgery, Sanador Hospital, Bucharest, ROU

**Keywords:** radiosurgical outcomes, interdisciplinary treatment approach, postoperative cerebrospinal fluid leak, sars-cov-2 neurological impact, frontotemporal neurosurgery, gender incidence disparity, genetic predisposition, monoclonal origins, meningioma pathophysiology

## Abstract

This case report elucidates the clinical and surgical journey of a 62-year-old patient with a history of multiple comorbidities including a severe acute respiratory syndrome coronavirus 2 (SARS‑CoV‑2) infection, presenting with temporospatial disorientation, bradypsychia, and bradyphasia, without motor deficits, diagnosed with sarcomatous meningioma and skull lysis. Amidst the complexities of managing primary brain tumors, this report underscores the significance of thorough morphopathological examination, while considering patient demographics and tumor localization in assessing the nature of the neoplasm. It highlights meningiomas as predominantly benign yet stemming from monoclonal proliferation, with their occurrence influenced by genetic predispositions and environmental factors such as ionizing radiation exposure. The intricate case details multiple surgical interventions necessitated by complications such as wound dehiscence and cerebrospinal fluid leaks, managed successfully through a tailored neurosurgical approach and meticulous postoperative care. This narrative reinforces the pivotal role of interdisciplinary collaboration, with substantial contributions from radiology, anesthesiology, intensive care, cardiology, infectious disease, and rehabilitation medicine in achieving favorable outcomes. The discussion contextualizes the patient’s condition within the broader neurosurgical literature, reflecting on the prognostic factors associated with giant meningiomas and the impact of factors like age and tumor location on resection outcomes. The case also delves into the efficacy of Gamma Knife radiosurgery in long-term tumor control, drawing on retrospective analyses. In conclusion, the case report advocates for a nuanced, individualized treatment, where the integration of multiple disciplines and responsive management of postoperative complications is critical to patient recovery. The successful resolution of this patient's condition exemplifies the quintessential nature of interdisciplinary collaboration and highlights the potential for optimizing neurosurgical protocols in the context of complex patient profiles.

## Introduction

When considering a primary brain tumor, we must first try and assess its character: whether it is malign or benign. Besides the morpho-pathological examination, which is of great importance, clinicians must take into consideration the age of the patient and the localization of the tumor. The clinical diversity of brain neoplasms is dominated by gliomas, which represent an impressive 75% of all primary malignancies that occur inside the brain [1,2]. When it comes to benign primary brain tumors, the most prevalent are non-malignant meningiomas [3].

Meningioma, although benign in approximately 95% of instances, invariably originates from a clonal proliferation that traces back to a singular progenitor cell. This neoplastic genesis is evidenced by cytogenetic analyses and array-comparative genomic hybridization (array-CGH) studies, which are congruent with the monoclonal origins observed in carcinomas [4]. The exact causes of meningiomas are uncertain because the incidence of this type of tumor is sporadic, appearing and disappearing through generations, families, and the general population. Epigenetically speaking, there seems to be a correlation between a high exposure rate to ionizing radiation and an increased probability of developing a type of meningioma, the incidence increasing if the individual has a genetic predisposition [5]. Fifty percent of patients with neurofibromatosis type 2 ( an inherited autosomal dominant disease) develop meningiomas. The predominant hypothesis for the development of meningiomas in the case of neurofibromatosis type 2 is a mutation in the NF2 gene, which is a powerful tumor-suppressing gene [6] that produces Merlin, a 595 amino acid protein

Moreover, the incidence of primary brain tumors is higher in the female than in the male population (15.80 vs. 14.33 per 100.000). The incidence is even higher when assessing the incidence of meningiomas (4.21 females vs 2.33 males per 100.000) [7].

## Case presentation

A 62-year-old individual presented with a complex medical and surgical history, including an operated right frontal intracranial expansive process, a recurrent and subsequently reoperated cerebrospinal fluid fistula, and a remitted severe acute respiratory syndrome coronavirus 2 (SARS‑CoV‑2) infection accompanied by seizure crises and intracranial hypertension syndrome. The patient also had a history of cerebellar abiotrophy syndrome, essential hypertension stage II with concomitant hypertensive retinopathy stage II.

Upon transfer from another hospital, the patient was diagnosed with a right frontal intracranial expansive process with associated bone lysis. The initial neurological examination revealed consciousness with cooperative behavior but also temporospatial disorientation, bradypsychia, and bradyphasia, with no evidence of paralysis. Advanced imaging techniques, including a native and contrast-enhanced brain MRI, identified a tumoral formation in the right frontal region. The lesion exhibited heterogeneous enhancement. Cerebral angiography further delineated the tumor's vascular supply and its mass effect, which resulted in a marked shift of the median cerebral axis.

**Figure 1:**
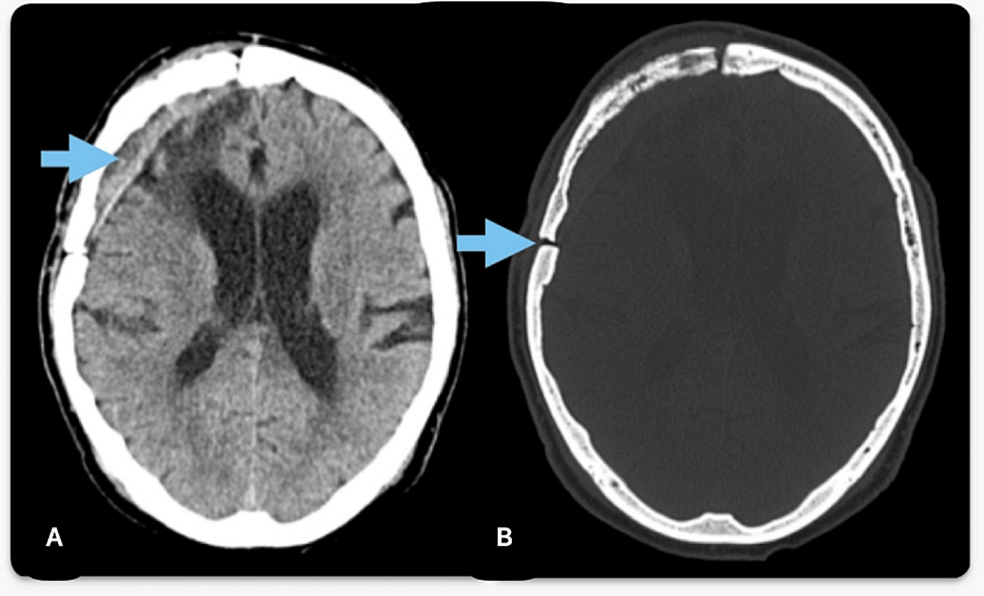
Postoperative CT scan aspect (A) CT tissue window highlights gross tumour resection; (B) CT bone window shows pterional flap on the right side of the skull

Given the severity of the presentation and after informed consent was obtained, a surgical intervention was performed, consisting of a paramedian pterional flap approach on the right side, through which a well-demarcated, moderately vascularized extraaxial tumor was completely resected (Figure 1).

**Figure 2:**
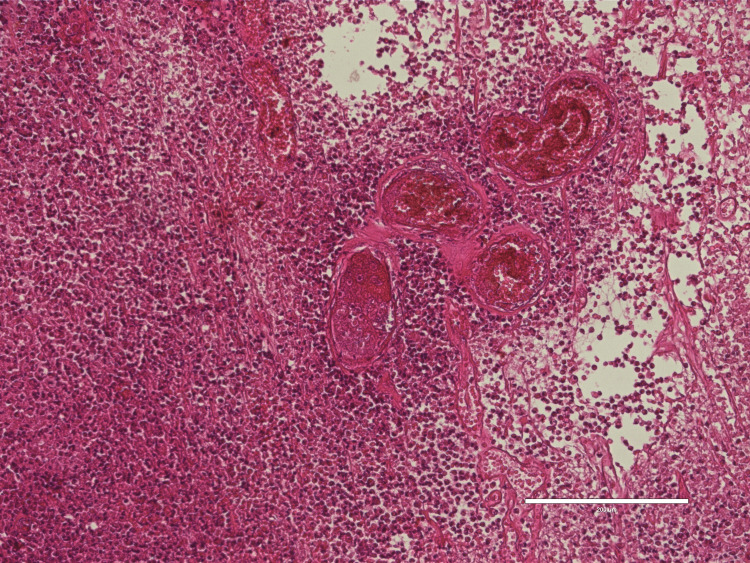
Histological aspect of anaplastic meningioma Image depicts the caracteristic sarcoma-like morphology of a anaplastic meningioma with an important mitotic count higher than 20/10 high-power fields, notably, significant vascularity can be observed within the tumoral process

**Figure 3:**
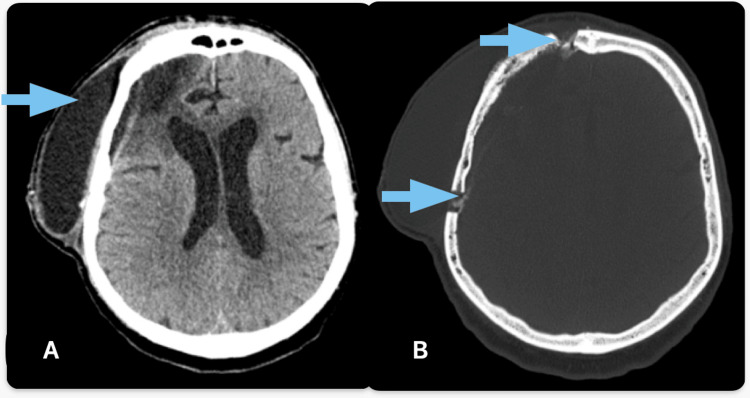
Postoperative dehiscence lesion Tissular window (A) of CT scan highlights the postoperative dehiscence lesion (blue arrow) at the level of pterional flap (B, blue arrows indicate trephine holes), shown on bone window

Histopathological examination revealed a sarcomatous meningioma, poorly differentiated (Figure 2). The immediate postoperative period was marked by favorable clinical outcomes. However, wound dehiscence (Figure 3) with cerebrospinal fluid leakage necessitated a second surgical procedure, which involved a dural plasty using temporal muscle fascia and repositioning of the bone flap with CranioFix. This intervention also followed a favorable clinical course.

Subsequently, a cerebrospinal fluid collection under the scalp was observed, leading to another informed surgical intervention. The procedure entailed reopening the previous surgical site, performing dural plasty, and securing the bone flap, culminating in a positive postoperative trajectory. The wound site was clear and no inflammatory signs were found. At discharge, the patient was conscious, cooperative, and without motor deficits (MRC = 5/5). During the hospitalization and the follow-up period, antiepileptics were administered and, therefore, comitial seizures weren't observed.

## Discussion

The clinical trajectory of this 62-year-old patient shows the complexities introduced by a plethora of pathologies in the management of intracranial neoplasms. The interplay between the patient's past intracranial surgeries, SARS-CoV-2 infection with neurological sequelae, and systemic conditions such as hypertension and discopathy necessitated a very specific and careful approach. These comorbidities potentially heightened the patient's risk profile, influencing both the surgical strategy and the anticipated postoperative course. The case prompts a re-evaluation of standard neurosurgical protocols, advocating for a more nuanced treatment paradigm that is responsive to individual patient histories. Recurrent intracranial lesions present a unique challenge; in this instance, the employment of a paramedian pterional flap approach for resection not only facilitated complete excision but also minimized disruption to the surrounding brain tissue, a technique tailored specifically for this particular case. 

Postoperative complications, particularly wound dehiscence and cerebrospinal fluid leak, were significant hurdles in the patient's recovery process. The management of these complications through additional surgeries, employing dural repair with autologous temporal muscle fascia and craniofixation for bone flap stabilization, illustrates the dynamic nature of postoperative care in neurosurgery. The effectiveness of these interventions reflects broader findings in neurosurgical literature, which advocate for early recognition and prompt, targeted management of post-surgical complications to optimize patient outcomes. 

Success in the resolution of this patient's intricate condition was not solely the purview of neurosurgical expertise but the result of a concerted interdisciplinary effort. Radiology was pivotal in providing high-resolution imaging that informed surgical planning. The perioperative and postoperative phases saw significant contributions from anesthesiology and intensive care, ensuring the patient's stability and recovery. The collaboration extended beyond the confines of the operating theater, with cardiology, infectious disease, and rehabilitation specialists playing critical roles, exemplifying the quintessential nature of interdisciplinary collaboration in managing complex neurosurgical conditions. Simpson Grading was not used in this case because it is a debated topic as to whether it is still considered accurate or not [8].

In a retrospective analysis of 80 patients with giant meningioma conducted by Narayan et al., they determined that a cohort of male patients, younger age groups, Simpson Grade 1 resection quality, tumors located at the skull base, employment of intraoperative navigation techniques, and World Health Organization (WHO) Grade 1 tumors correlate with an enhanced prognostic outlook [9].

Another study conducted by Quiñones-Hinojosa et al. assessed the pre-operative factors suggestive of a possible total resectability of giant intracranial meningiomas [10]. In this study, 58% of patients, totaling 39 individuals, achieved gross total resection (GTR) classified as Simpson grades I or II. There were no instances of perioperative mortality recorded. At the final follow-up, there was an improvement in symptoms in 58% of patients, no change in 30%, and a deterioration in 12%. The multivariate analysis for all patients with large meningiomas indicated that being over the age of 45 years (OR 0.127, 95%CI 0.026-0.616, p=0.01) and involvement of the superior sagittal sinus (OR 0.160, 95%CI 0.026-0.976, p=0.05) were associated with a decreased likelihood of achieving GTR. Conversely, preoperative embolization (OR 8.087, 95%CI 1.719-38.044, p=0.008) correlated with a higher likelihood of GTR. Specifically, for supratentorial meningiomas, involvement of the superior sagittal sinus (OR 0.077, 95%CI 0.010-0.571, p=0.01) and preoperative embolization (OR 10.492, 95%CI 1.961-56.135, p=0.006) were found to be independent predictors of successful GTR [10]. 

A retrospective analysis by Lippitz et al. reports a decade-long follow-up, representing one of the most extensive temporal assessments of meningiomas treated via radiosurgery currently available [11]. After the treatment utilizing Gamma-Knife, 108/123 meningioma tumors maintained tumor control after 10 years. This series substantiates the sustained efficacy of Gamma Knife therapy in achieving high rates of local tumor control, reinforcing the reliability of this adjuvant treatment in meningioma.

## Conclusions

This case highlights the importance of teamwork when taking into consideration these kinds of complex pathologies. Collaboration between different fields of medicine is as important in this case as careful surgical planning. Thanks to careful postoperative monitorization, the dehiscence that later occurred was rapidly treated, resulting in an overall good recovery.
